# Novel three-dimensional bone ‘mapping’ software can help assess progression of osseous metastases from routine CT

**DOI:** 10.1186/s13014-017-0880-2

**Published:** 2017-08-30

**Authors:** D. Thurtle, G. M. Treece, T. Barrett, V. J. Gnanapragasam

**Affiliations:** 1 Academic Urology Group, Department of Surgery, University of Cambridge, Box 279, Cambridge Biomedical Campus, Cambridge, CB2 0QQ UK; 2 Cambridge University Hospitals NHS Foundation Trust, Hills Road, Cambridge, CB2 0QQ UK; 3 Medical Imaging Group, Department of Engineering, University of Cambridge, Trumpington Street, Cambridge, CB2 1PZ UK; 4 Department of Radiology, University of Cambridge School of Clinical Medicine, Hills Road, Cambridge, CB2 0QQ UK

**Keywords:** Bone metastases, Imaging techniques, Medical imaging, Prostate cancer, Computed tomography, 3-D imaging

## Abstract

Imaging of bone metastasis response to therapy is a research priority. Stradwin is a new software-tool, with demonstrated sub-voxel accuracy in assessing cortical bone properties from routine CT. We applied this technology to the context of osseous metastases, with particular focus on disease progression using prostate cancer as a model. 3D–rendered ‘bone-maps’ were produced for 20 men with advanced prostate cancer, including a sub-cohort of 9 who had undergone serial scans. Correlation between baseline interpretation and assessments of progression between modalities was assessed. Bone-maps took significantly less time to interpret than CT bone windows (*P* < 0.001). Initial bone-mapping, without adjustment, demonstrated sensitivity and specificity for suspicious areas on CT of 70.7% and 73.1% respectively. Evaluating disease over time, concordance between bone-maps and current practice using RECIST outcomes was 100%.

This study demonstrates the feasibility and potential use of this free post-processing software in the serial assessment of osseous metastases.

**Electronic supplementary material:**

The online version of this article (10.1186/s13014-017-0880-2) contains supplementary material, which is available to authorized users.

## Introduction

Bone scintigraphy (BS) remains the workhorse for detection of bone metastases in many cancer types including prostate cancer (PCa). However, BS has significant limitations in assessing changes to osseous metastases over time, particularly due to ‘flare’ effects and low specificity [1, 2]. BS also fails to provide any additional information on soft tissue or nodal metastases. With the improving efficacy of bone-targeting therapies, such as radium 223 [3], the evaluation of bone metastasis response to therapy is of growing importance, and has been made a Europe-wide research priority [4]. Positron-emission tomography techniques have shown promise in this setting [4, 5], but remain expensive and often inaccessible outside of the trial setting. As a result, computed tomography (CT) is increasingly used in the serial assessment of disease over time.

Novel post-processing software developed in Cambridge (Stradwin) has been demonstrated to accurately assess bone properties using routine CT. The technique, which has been described previously, uses a complex model-based fit approach, calculated from thousands of data-points across the bone surface assessed by semi-automatic segmentation [6]. Accurate cortical bone thickness estimates have been demonstrated down to 0.3 mm in cadaveric samples and the technique has been validated in vivo in the context of osteoporosis and hip fracture [6, 7]. Here, we assessed the feasibility and application of this software to assess trabecular bone density in osseous metastases, using PCa as a model. Our particular focus was on the comparison of disease burden over time.

## Materials and methods

Following institutional approval (CUH/ID6669) routine abdomino-pelvic CT scans of 20 patients with metastatic PCa were retrospectively retrieved, anonymised at source, and processed using Stradwin 5.1 software.^1^ 3-dimensional rendered ‘bone-maps’ of the pelvis and lower vertebral column were produced for each patient, with trabecular bone attenuation coefficients ‘mapped’ to the surface (Additional file 1). Contemporaneous isotope BS (within 2 months of CT), were also retrieved. A sub-cohort of 9 men who had undergone follow-up CT and contemporaneous BS, during the study period, were selected for further study. The CT scans of these 9 men were also retrieved and processed.

Bone-maps and the bone-windows of original CT scans were randomised and reviewed independently by a consultant radiologist blinded to previous imaging or clinical information. The radiologist localised suspicious areas of osseous metastasis using a novel validation tool (Additional file 1: Figure S1). Time taken to interpret each modality was recorded. Results for each of the skeletal areas isolated on the validation tool were compared between each modality to calculate comparative sensitivity and specificity, initially comparing bone-maps to CT. Data management and analysis was performed in MS Excel (Washington, USA) and StatsDirect (Altrincham, UK) respectively.

Comparing baseline and follow-up bone maps, osseous disease stability, progression or response was assessed using RECIST outcomes [8]. These outcome decisions were compared against those made by using the ‘gold standard’ of current clinical practice, using BS and CT in combination to assess disease progression or response.

## Results

The median (IQR) age across the cohort of 20 men was 73.5 (69.5–76.25) years. Median (IQR) PSA at diagnosis was 130.5 (26–389) ng/mL. 10 men had a clinical diagnosis of PCa. Of the remainder, 5 had histological Grade Group 5 PCa (Gleason 9 or 10) at diagnosis, 3 had Grade Group 4 (Gleason 8), and 2 had Grade Group 2 (Gleason 3 + 4). All men had at least one bone metastasis demonstrated on both BS and the imaged skeleton on CT.

**Fig. 1:**
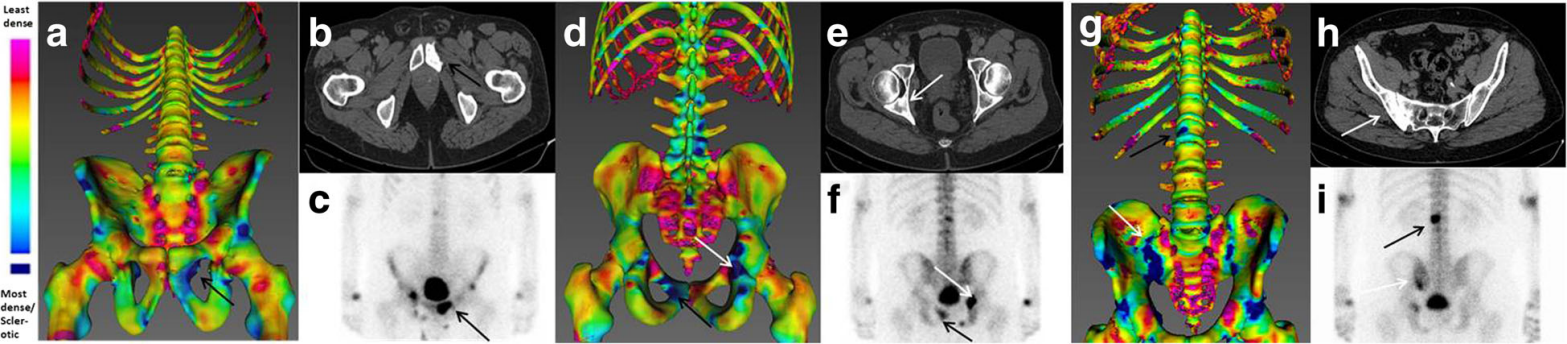
79-year old male with Grade Group 5 (Gleason 5 + 4) prostate cancer. Anterior (**a**) and posterior **d** stills of a 3D bone map with trabecular bone attenuation coefficients mapped to the surface (legend left of image **a**). Anterior (**c**) and posterior (**f**) isotope bone scans and axial CT images (**b** and **e**) are shown from the same date. Dark blue areas on the bone maps correspond to areas of sclerosis, well-demonstrated in the left pubis (black arrows **a**, **b**, **c**, **d** & **f**) and right acetabulum (white arrows **d**, **e** & **f**). Anterior view of the same patient’s 3D bone map 24 months later (**g**) demonstrates particular progression in the right ilium (white arrow) and L2 vertebra (black arrow), also demonstrated on the original CT(**h**) and contemporaneous BS(**i**)

CTs contained a mean (±SD) 281 (±53) images whereas single 3D–rendered bone-map overviews were produced for each case (e.g., Fig. 1a). Accordingly, bone-maps took significantly less time (mean (±SD) 93.6 s (±29.5)) to interpret than CT bone windows (217.3 s (±63.1) (*p* < 0.001).

Interpretation of bone-maps led to calls of 235 areas appearing malignant and 47 suspicious, compared to 264 and 28 respectively for CT. Without any software adjustments, the sensitivity of bone-maps for demonstrating malignant areas recorded from CT was 70.7%, with 73.1% specificity. Correlation between CT and BS interpretation was also assessed, with CT demonstrating 85.6% sensitivity for metastases reported on BS with 64.7% specificity (Table 1).

**Table 1:** Interpretation time, results and sensitivity/specificity for each modality against the reported comparator

Test modality	Mean interpretation time (s)	Malignant areas	Suspicious areas	Compar-ator	Sensitivity (Exc ‘sus’)	Specificity (Exc ‘sus’)	Sensitivity (Inc ‘sus’)	Specificity (Inc ‘sus’)
Bone Map	96.3	235	47	CT	70.71	73.08	69.05	58.28
CT	222.1	264	28	BS	85.63	64.71	81.56	62.89

These were assessed including or excluding calls of suspicious (‘sus’) areas

The median (range) interval between processed CTs in the sub-cohort assessed for progression was 13 (4–23) months. The mean (range) time between CT and BS in this cohort was 4.1 (0–25) days. 7 of these 9 patients were reported to have progressive disease using CT and BS in combination, the other 2 had stable disease. Using bone-maps in isolation the same results were reported, with the same patients reported to have progressive and stable disease respectively. This equates to 100% concordance within our cohort, between bone-maps and the current clinical standard for assessing disease progression.

## Discussion

Applying software designed for alternative purposes we have demonstrated proof-of-concept for the use of Stradwin to non-invasively assess bone metastases. Using a simple post-processing step, CT data can be transformed in to easily-interpreted single-visualisation 3D–overviews of osseous disease burden. These single image bone maps took significantly less time to read, and may be easier for non-specialists to interpret.

The relatively low values for sensitivity and specificity of bone maps compared to CT should be viewed in the context of only marginally better results when CT was compared to BS. This may be due, in part, to the artificial situation of viewing the modalities in complete isolation from one another, though it is also acknowledged an element of subjectivity exists with any imaging interpretation. Without histological correlation we are unable to conclude that areas only reported as malignant or suspicious on bone-maps are indeed false positives, rather this may reflect inadequacies of our reference modalities.

The technique shows particular promise for comparisons of disease over time, with 100% concordance in our cohort. The potential clinical utility of assessing treatment response is significant given the increasing efficacy of bone-targeting therapies, inadequacies of BS in this context, and high costs of functional imaging techniques such as PET-CT. Although we have focussed here on assessments of overall disease progression, the technology may also be effective in assessing individual bone metastasis size and response. Evaluating bone metastases in combination with the software’s validated accuracy in assessing cortical properties may also be of significant clinical value in the assessment of pathological fractures.

There are numerous potential advantages to this technology. CT scans are cheap, quick and widely available; most patients with cancer will already undergo a baseline CT scan and as such no additional radiation would be required. This post-processing software is free-to-use, intuitive and fully compatible with DICOM data. The produced single-image overviews (e.g., Fig. 1) would be of value in the multi-disciplinary team meeting or as a discussion-aide when counselling patients about disease extent, progression or stability. Bone-maps may also prove useful adjuncts for radiologists, highlighting areas to particularly focus upon. This technology would have similar potential in other cancers that preferentially metastasise to the bone e.g. breast cancer.

Potential drawbacks include that current processing is not fully-automated, allowing no standardisation of colour thresholds between scans, or patients. This processing step does also require a small amount of additional time. However, it is anticipated that optimisation of this software specifically for use in osseous metastasis could allow a fully automated, standardised processing protocol. Other prospective software amendments seek to set thresholds such that only abnormally sclerotic bone is highlighted, removing the background ‘noise’ in the final bone maps. Currently, bone-maps require visual interpretation with the associated subjectivity herewith. Eventually we anticipate that computerised quantification of abnormal areas from these bone maps may enable estimates of total skeletal involvement. Such a numerical value would be invaluable in assessing disease progression or response to treatment, and remove the need for visual interpretation of these 3-dimensional constructs. This could have applications in therapeutic trial design in addition to routine clinical practice.

Limitations exist in terms of study design – with a retrospective format, lack of a histological gold standard and limited numbers. It is intuitive that this technique, using routine non-functional CT, relies upon architectural changes within the bone. For this reason, when assessing disease over time against RECIST outcomes it is unlikely bone maps could demonstrate complete or partial response, as burnt-out metastases typically remain sclerotic. For this reason, functional imaging modalities may be more sensitive earlier in the metastatic process or when assessing for early response or non-response to treatment.

In summary we report here a potential quick and cost-effective method for assessing bone metastasis response. These promising preliminary results justify further work on this exciting free technology which we encourage other institutions to consider trialling as we seek to optimise and improve its value in this important setting.

## Additional file

Additional file 1: Instructional methodology to create 'bone maps' using Stradwin software and validation tools for skeletal metastases. (DOCX 258 kb)

## Footnotes

1 Stradwin is available as a free download from http://mi.eng.cam.ac.uk/~rwp/stradwin/
