# Supplementary material for: Novel three-dimensional bone ‘mapping’ software can help assess progression of osseous metastases from routine CT
Source: Radiat Oncol. 2017 Aug 30;12:143. doi: 10.1186/s13014-017-0880-2 (PMC5577835; doi:10.1186/s13014-017-0880-2)
Supplement: Additional file 1: — Instructional methodology to create 'bone maps' using Stradwin software and validation tools for skeletal metastases. (DOCX 258 kb) [file 13014_2017_880_MOESM1_ESM.docx]

**Radiation Oncology Supplementary Files**

**Methodology to create a ‘Bone Map’ using Stradwin 5.2**

Stradwin is available as a free download from <http://mi.eng.cam.ac.uk/~rwp/stradwin/>

Open a DICOM file using ‘Open’ and selecting the relevant file.

**Draw tab**

Select ‘Task’ and ‘Draw’

On the draw tab click the box beside ‘use threshold markers’

Slide the tab beneath, such that the majority of the bones are pink, but very little else is coloured.

In the sliding tab where it says ‘each 0-100 frames’ select 1. (You can use the keyboard if the mouse cannot choose 1)

Use the pencil tool to click on a single area of bone. All attached areas will become selected.

Select ‘update all’ above the thresholds section.

Standard settings in the ‘surface resolution area should be:
surface resolution ‘medium’

Smoothing type ‘standard’

Smoothing strength ‘very low’

UNCLICK ‘use threshold sliders’ before moving to the thickness tab

**Thickness tab**

Select ‘line length for thickness estimates (mm) as: 18

Tick ‘inner’ and ‘outer’ boxes beneath

‘Estimate cortical density’ can be used to set values for inner density (will take a few minutes)

Select ‘cortical thickness mapping’: Current surface… select ‘Object 0’.

Select ‘Map thickness over surface’ (will take a few minutes)

On the right-hand tab ensure colour with ‘trabecular (HU)’ is selected

Using ‘pink-yellow-green-blue’ is the standard colour scheme, others can be used.

Adjust the ‘max density’ and ‘smoothing’ as necessary

Save to PLY format, this can be accessed by most 3D reading software.

NOTE: The settings outlined above are those that have been used in this publication, but can all be altered and experimented with.

**
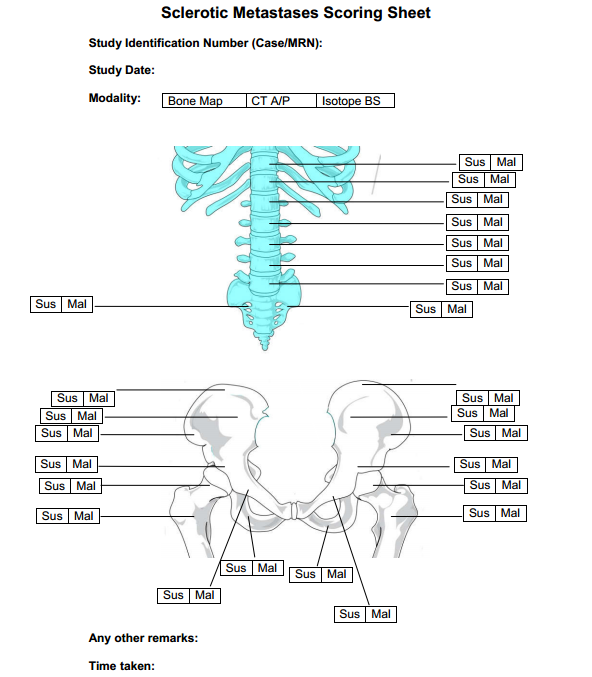
**

Figure S1: Novel validation tool used to isolate areas suspicious ('sus') or malignant ('mal') for bone metastasis from the modality used.

**
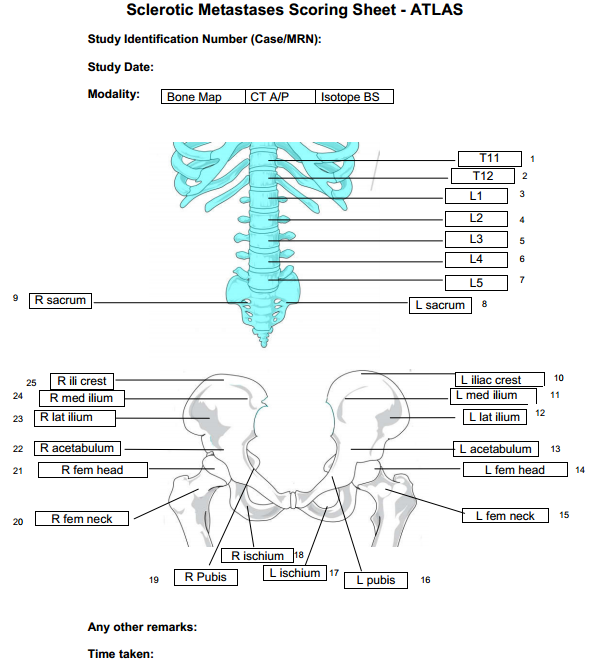
**

Figure S2: Atlas to the novel validation tool (Figure S1). Each anatomical region is numbered, for easy comparison between modalities.
